# 10 Strategies for prevention of perineal wound dehiscence after intersphincteric proctectomy for perianal Crohn’s disease

**DOI:** 10.1007/s10151-026-03356-y

**Published:** 2026-05-25

**Authors:** Stefan D. Holubar, Phil Tozer

**Affiliations:** 1 Department of Colon and Rectal Surgery, Cleveland Clinic, Cleveland Clinic Lerner College of Medicine and Case Western Reserve University, 9500 Euclid Ave, A30, ClevelandCleveland, OH 44122 USA; 2 Department of Colorectal Surgery, St Mark’s Hospital and Academic Institute, London, UK

**Keywords:** Crohn’s disease, Perianal fistulae, Proctectomy, Perineal wound dehiscence, Presacral sinus, Omental pedicled flap, Omentoplasty, Incisional negative pressure wound therapy

## Abstract

Non-healing perineal wounds (NHPWs), including persistent presacral sinuses, remain a common and morbid complication after elective intersphincteric proctectomy (ISP) for fistulizing perianal Crohn’s disease (pCD), with reported healing rates of approximately 70% at 1 year. These complications significantly impair quality of life and increase healthcare utilization, highlighting the importance of prevention. This narrative review summarizes contemporary evidence and expert practice to present ten pragmatic strategies to reduce perineal wound dehiscence after ISP for pCD. Prevention begins with comprehensive preoperative optimization, including correction of malnutrition and anemia, minimization of corticosteroid exposure, smoking and nicotine cessation, and optimization of relevant medical comorbidities. Careful assessment and control of Crohn’s disease activity are emphasized through current disease staging, proactive therapeutic drug monitoring, and aggressive surgical control of perianal sepsis. These measures often involve repeated examinations under anesthesia, drainage of abscesses and fistula tracts, fecal diversion when indicated, and selective use of hyperbaric oxygen therapy to improve local conditions before definitive surgery. Operative strategies focus on technical decisions that minimize perineal wound burden and promote healing. Key elements include performing proctectomy in the intersphincteric plane with total mesorectal excision when feasible, meticulous debridement and management of fistula tracts, and robust multilayered perineal closure. Adjunctive techniques such as horizontal mattress suturing of the levators to enhance tissue apposition, use of pedicled omental flaps with fluorescence angiography to confirm perfusion, and prophylactic incisional negative pressure wound therapy are highlighted. Collectively, these strategies provide a practical framework to reduce perineal wound complications after ISP for pCD.

## Introduction

**Fig. 1:**
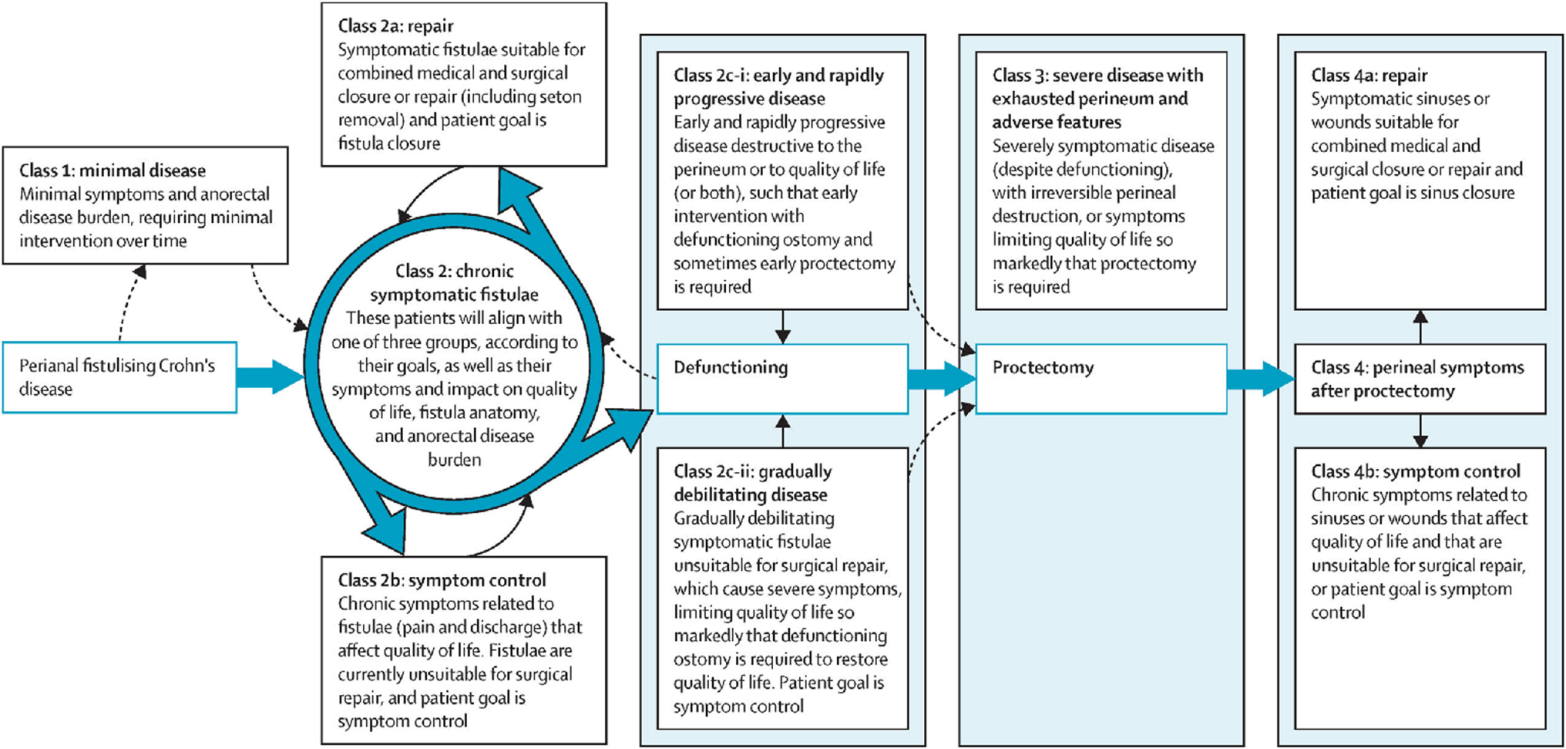
Treatment optimization and classification (TOpClass) consortium classification of perianal Crohn’s disease. Reproduced with permission from the TOpClass Consortium (https://topclass-ibd.com/)

Fistulizing perianal Crohn’s disease (pCD) may require elective intersphincteric proctectomy (ISP) in up to 20% of patients, classified as TOpClass Consortium Class 3 pCD (Fig. 1) [1, 2]. After ISP for pCD, many patients develop non-healing perineal wounds (NHPW), also known as persistent presacral sinuses, classified as Class 4 pCD, with only 70% of pCD patients fully healed at 12 months [3, 4]. Patients with other forms of inflammatory bowel disease (IBD) may also require ISP, and NHPWs are notably lower at approximately 20% in ulcerative colitis (UC) or after pouch excision [5–8].

Many mitigation strategies have been reported, a testament to the recalcitrant nature of these wounds, emphasizing the need for prevention strategies to optimize outcomes [9]. In this Short Report, we review preoperative optimization and surgical techniques to prevent class 4 pCD.

### Preoperative optimization

ISP is often the last step in the pathway of treatment failure; however, these steps (Table 1) are critical for optimizing pCD patients for ISP. Preoperatively, modifiable risk factors for NHPWs are optimized [10].

**Table 1:** Perineal wound dehiscence prevention strategies in Crohn’s disease

Strategy	Description
1. Preoperative optimization	Malnutrition: nutrition consult; high-calorie, high-protein nutritional supplements (shakes) Corticosteroids: weaning off or to as a low dose as possible Anemia: intravenous iron supplementation Nicotine cessation: cognitive behavioral therapy, pharmacotherapy, nicotine-free vapes Comorbidities: optimization of medical comorbidities such as diabetes
2. Crohn’s disease monitoring and optimized advanced therapy for Crohn’s disease	Disease staging: up-to-date endoscopy and cross-section CT or MR enterography, laboratory reports (comprehensive metabolic panel, complete blood count, C-reactive protein, fecal calprotectin) Therapeutic disease monitoring: Use of drug levels and anti-drug antibodies
3. Drainage of all abscesses and fistula tracts	Liberal use of preoperative MR perineum, EUAs with wide drainage, mushroom drains, and setons
4. Diversion and/or hyperbaric oxygen therapy before proctectomy	Allows perianal sepsis to settle. Patients’ refractory to diversion may benefit from hyperbaric oxygen therapy
5. Intersphincteric proctectomy	If possible, the proctectomy is performed in the intersphincteric plane to minimize the defect and spare the local muscles for reapproximation of the levators and sphincters
6. Debridement and closure of all fistula tracts	All granulation tissue of all tracts are debrided and internal openings of the tracts suture ligated, or fistulotomies performed to lay them open. Fistulae and inflamed tissue do not need to be excised
7. Perineal defect closure	The perineal defect is closed in multiple layers of absorbable suture. Mattress sutures for the levators allows for bulky midline tissue reapproximation and offloads the suture tension from the center of the wound to the sides where there is healthy, bulky muscle
8. Omental pedicled flap with fluorescence angiography perfusion assessment	A pedicled flap based on one of the gastroepiploic pedicles. Confirmation of perfusion to the distal flap may be confirmed with indocyanine green fluorescence angiography
9. Prophylactic incisional negative pressure wound therapy	After closure of the perineal skin, a short-term (1-week) incisional negative pressure wound therapy device may be applied. Alternatively, the skin may be left open and a traditional wound vac placed. Importantly, stoma adhesive (karaya) paste is used to obtain a seal in the intergluteal cleft
10. Early wound treatment of perineal wound dehiscence	Early, aggressive, multimodal treatment of perineal wound dehiscence and/or surgical site infections to prevent wound fibrosis and epithelization

*CT* computed tomography, *MR* magnetic resonance, *EUA* examination under anesthesia

### Medical optimization

Malnutrition: Initial assessment confirms whether patients have or are at risk for perioperative nutritional impairment [11, 12]. Patients with malnutrition, a negative nitrogen balance, a catabolic state marked by recent unintentional weight loss, and abnormal nutritional serum markers are referred for formal nutritional assessment. Sarcopenia, in which patients are both malnourished and overweight, has emerged as a strong risk factor for complications after surgery for IBD [13, 14]. For pCD with intestinal stricture(s), exclusive enteral nutrition (EEN) with high-calorie, high-protein shakes is often recommended, reserving total parenteral nutrition for patients who cannot (short gut) or should not (obstruction) tolerate EEN [15].

Corticosteroids: Elective ISP is typically deferred until corticosteroids are tapered off to avoid steroid-induced wound non-healing [7]. Limited data suggest that if corticosteroids cannot be weaned, a short course of supplemental vitamin A (due to hepatotoxicity with long-term use) may help overcome steroid-related impaired collagen synthesis [16, 17].

Anemia: Anemia is independently associated with an increased risk of postoperative complications after surgery and is common in patients with IBD, seen in up to 50% of patients preoperatively [18]. Intravenous iron supplementation may quickly replace depleted iron stores preoperatively [19].

Nicotine cessation: Smoking, vaping, and other nicotine-containing products are risk factors for postoperative complications. Nicotine cessation strategies include cognitive behavioral therapy, pharmacotherapy, and nicotine-free vapes.

Comorbidities: Medical comorbidities, such as diabetes and other systemic diseases, can negatively affect postoperative healing rates and should be optimized preoperatively in a multidisciplinary manner.

### Crohn’s disease treatment optimization

Disease staging: CD staging should be current as of ISP. Up-to-date endoscopy and cross-sectional CT or MR enterography are performed [20, 21]. Laboratory tests (comprehensive metabolic panel, complete blood count, C-reactive protein, and fecal calprotectin) are used to assess the sequelae of inadequate response to ongoing therapy in a treat-to-target manner [22].

Therapeutic disease monitoring: Advanced therapy should be optimized before elective ISP in patients with severe class 3 pCD. We favor proactive therapeutic drug monitoring (when available) using serum drug concentrations and anti-drug antibody levels, combined with other objective measures of response to medical therapy [23, 24].

**Fig. 2:**
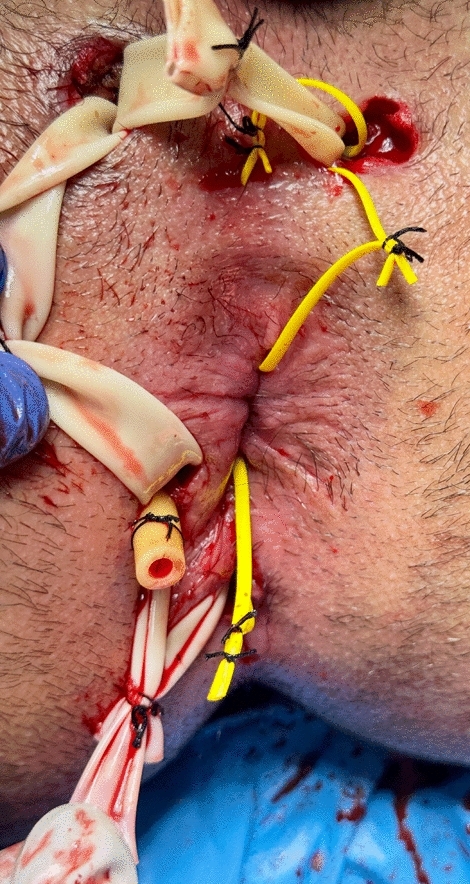
Intraoperative photograph of severe fistulizing perianal Crohn’s disease depicting drainage of all collections and tracts with Penrose drains (upper left and anteriorly), mushroom catheters (bottom left), and draining setons (right, anterior). © Cleveland Clinic Foundation 2025

Surgical rationalization of perianal sepsis: Patients with active pCD require a combined medical-surgical approach, with surgical interventions focused on the liberal use of preoperative magnetic resonance imaging (MRI) of the perineum to guide therapeutic, often repeated examinations under anesthesia (EUAs) with aggressive and meticulous drainage of all abscesses and fistula tracts using Penrose drains, mushroom catheters, and draining setons (Fig. 2).

Patients’ refractory to attempts at maximal local surgical drainage and whose advanced therapy is optimized, designated as Class 2c pCD, often undergo fecal diversion with a diverting loop ileostomy or colostomy to allow the perianal sepsis to settle and “cool off” before elective ISP. Unfortunately, for most (80%), “temporary” diversion is permanent and should be presented as such by clinicians [25]. This allows for more aggressive drainage and laying open of tracts where sphincter sacrifice is necessary.

Hyperbaric oxygen therapy: When severe pCD is recalcitrant to optimized advanced therapy and fecal diversion, hyperbaric oxygen therapy may improve local conditions in preparation for ISP [9, 26, 27].

## Surgical techniques

### Intersphincteric dissection

**Fig. 3:**
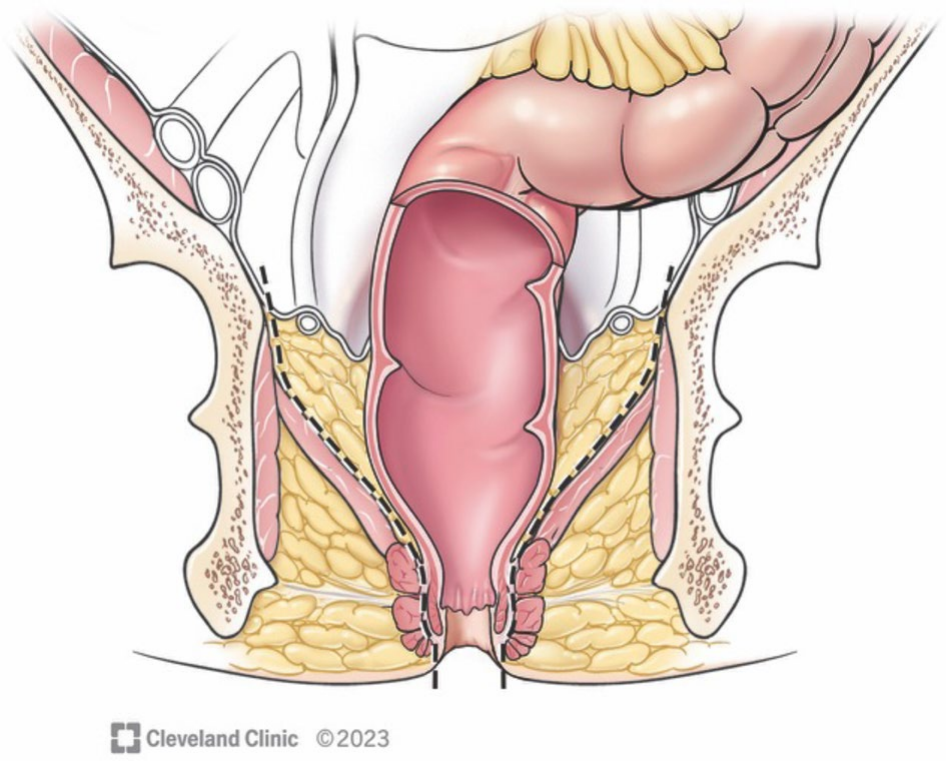
Illustration of the plane of intersphincteric dissection during completion proctectomy for inflammatory bowel disease. © Cleveland Clinic Foundation 2025

Most patients with IBD who undergo completion proctectomy are candidates for intersphincteric dissection (Fig. 3), as opposed to abdominoperineal resection for rectal adenocarcinoma or close rectal dissection in restorative proctectomy in UC. The abdominal portion of the completion proctectomy is first performed, often in a minimally invasive manner, and typically as a total mesorectal excision (TME). In 2019, de Groof et al. showed a significantly lower rate of NHPW (17.6% vs. 59.5%, *p* = 0.007) after TME than after intramesorectal dissection for ISP in CD [28].

**Fig. 4:**
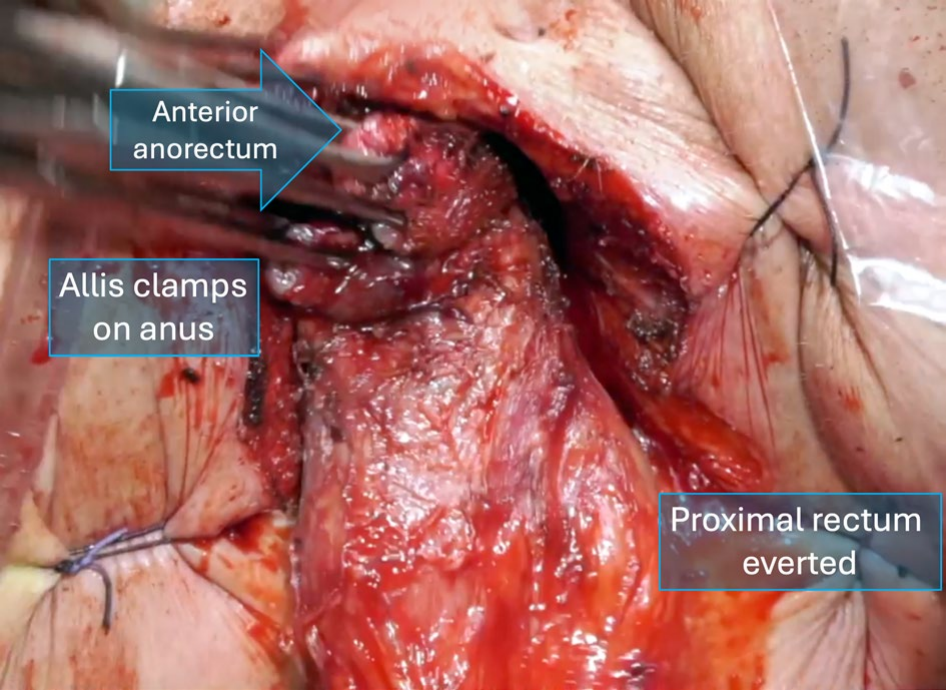
Eversion of the proximal rectum, mid-rectum, and mesorectum posteriorly in a “candy-cane” manner facilitates completion of the dissection of the anterior anorectum off the posterior wall of the vagina or prostate. © Cleveland Clinic Foundation, 2025

After the rectum is mobilized transabdominally, perineal dissection is performed. Obtaining optimal perineal exposure is crucial; ISD is typically performed in the high lithotomy position, assuring that the anus and perineum are positioned off the lower edge of the bed and raised with a bolster beneath the pelvis. Optimal anal exposure is obtained using a Lone-Star retractor system, although we prefer anal eversion sutures using heavy sutures placed in four positions to efface the anus (Fig. 4). The ISD begins with electrocautery to resect all anoderm (typically red-purple), which, if left behind, may result in a moist perineum in the long term. The intersphincteric groove is identified by palpation with a retractor stretching the IAS, and the incision proceeds circumferentially in the intersphincteric plane between the internal and external anal sphincters, identifying the longitudinal muscle of the anus, coming across fistula tracts in the process, which are transected, not excised.

**Fig. 5:**
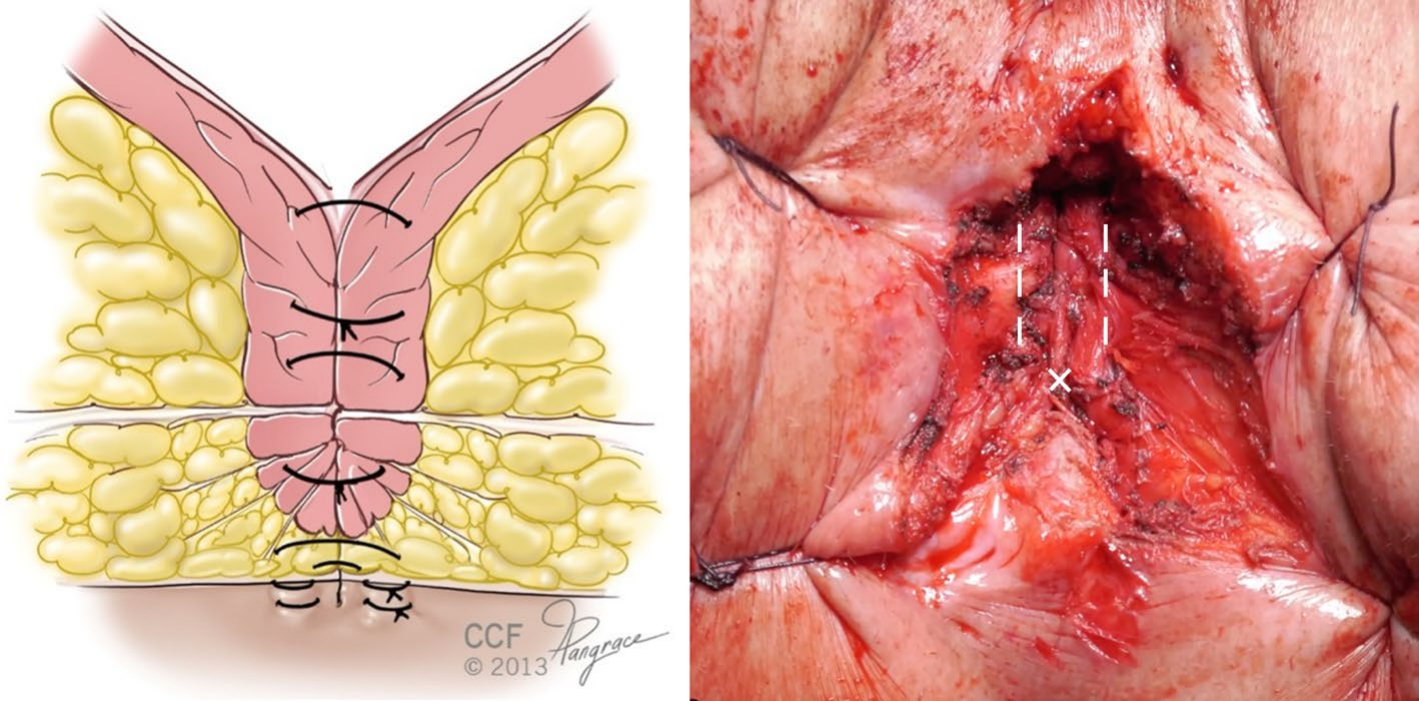
Reapproximation of the levators using heavy horizontal mattress sutures after proctectomy for fistulizing perianal Crohn’s disease. Illustration of overall approach (left panel) and intraoperative photo (right pane); note the anal eversion sutures laterally; the annotated white lines demonstrate the absence of sutures in the upper midline compared with a single figure-8 suture crossing the midline inferiorly. © Cleveland Clinic Foundation, 2025

The dissection proceeds posteriorly and then bilaterally; the anterior dissection is typically performed last to aid in the dissection of the anorectum off the vagina or prostate. The intersphincteric plane is bloodless, but bleeding from the EAS is common, and bleeding points are meticulously electrocauterized when encountered to avoid vessel retraction and blood obscuring the operative field and plane of dissection. The anococcygeal ligament is transected, and the distal extent of the pelvic dissection is reached, often with an anterior direction of incision, releasing the pneumoperitoneum. The dissection proceeds bilaterally, often with a finger hooking around the levator, and the abdominal team moves the mobilized rectum to aid in dividing the levator close to the rectal wall. The rectum may be eviscerated posteriorly in a “candy-cane” maneuver (Fig. 5) to facilitate visualization of the posterior wall of the vagina or prostate, and the anterior dissection is completed. In women, this may be aided by digitalization of the vagina to identify the posterior vaginal wall. After the specimen is passed off the field, the perineal wound may be irrigated with chlorhexidine solution followed by saline, and meticulous hemostasis is assured.

### Method of perineal wound closure

Before perineal wound closure, draining setons are removed, granulation tissue of all tracts is debrided, internal openings of the tracts are suture ligated, fistulectomy with excision of the tract or fistulotomies are performed to lay them open. Fistulectomies and fistulotomies may be used liberally as postoperative incontinence is not a concern. The perineal defect is closed with at least four layers (levator, external sphincter, subcutaneous tissue, and skin) of absorbable sutures. We typically use a heavy (#1 or 0—0) braided absorbable suture with a large (CT-1) needle for levator reapproximation, traditionally accomplished using figure-8 (FOE) sutures. However, in IBD, closing the levators with horizontal mattress sutures has several theoretical and practical advantages over FOE (Table 2) as they allow for bulky midline tissue approximation and offload the suture tension from the center of the wound to the sides where there is healthy, bulky tissue that is less likely to have been affected by fistula tracts and fibrosis (Fig. 4).

**Table 2:** Comparison of the biomechanical features of figure-8 and horizontal mattress sutures for closure of the levators after completion of proctectomy with intersphincteric dissection for inflammatory bowel disease

Feature	Figure eight	Horizontal mattress suture
Main vector	Diagonal (oblique) centripetal pull towards central focal cinch point at the center of the wound where the knot is tied	Transverse tension-band with symmetric pull across both levators
Dead-space closure	Focal 3-dimensional cinching the center of the wound; cinching effect attenuates radially away from center	Broad-based vertical muscle-to-muscle tissue approximation with effective midline leveling of levators
Apposition/eversion	Point-dominate apposition with minimal eversion	Even apposition along the bite, strong lateral tension bands along the levators
Tissue pressure	Concentrated centripetal compression force (“crush zone”) at central crossing/knot	Distributed compressive forces across a wider muscle segment
Midline result	Visible/palpable X (knot) in midline	Clean midline with bulky, well-approximated levator; knots kept lateral

### Omental pedicled flaps

**Fig. 6:**
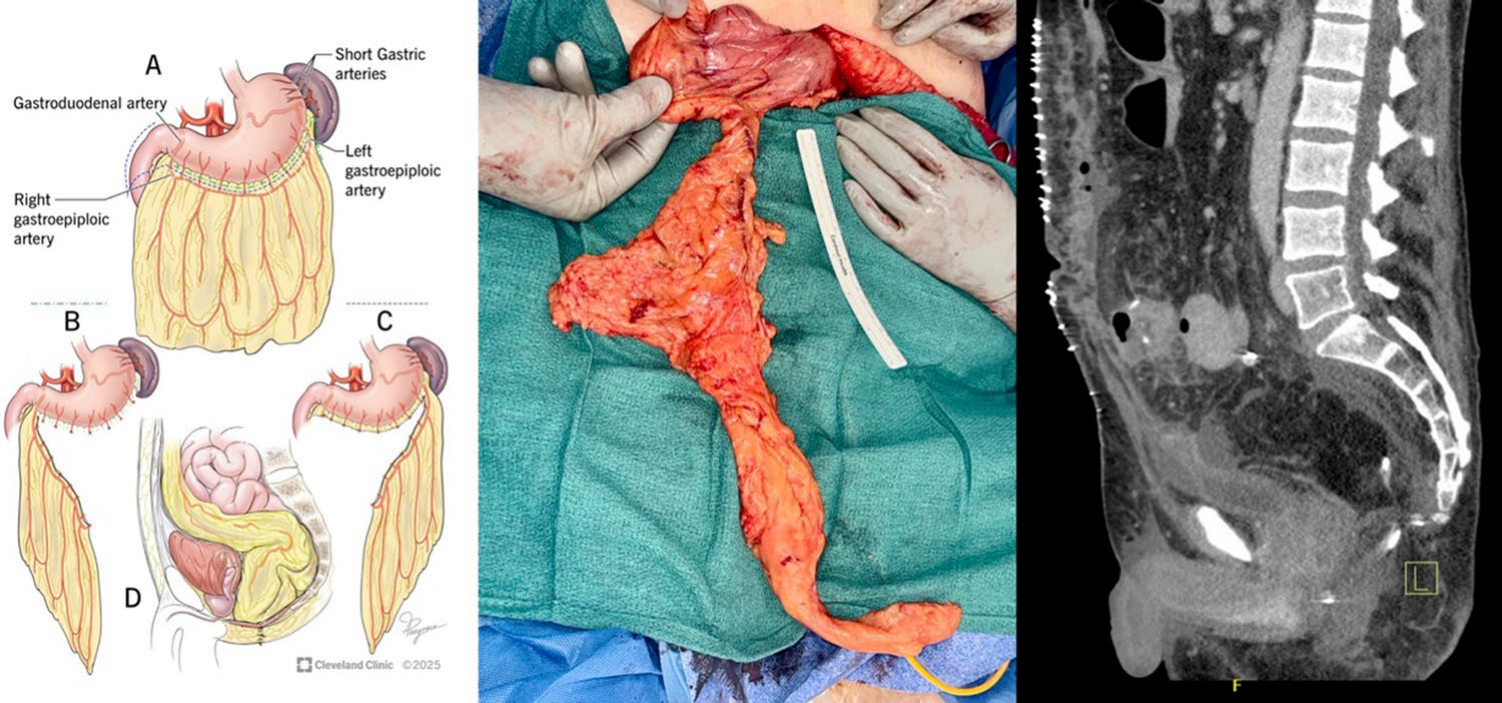
Technique for a pedicled omental flap (OPF) based on the right or left gastroepiploic artery (left panel); an OPF based on the right gastroepiploic artery after high ligation of the left gastroepiploic artery in a patient with prior subtotal omentectomy undergoing pouch excision (middle panel); postoperative computed tomography (CT) appearance of an OPF filling the pelvis (right panel). © Cleveland Clinic Foundation, 2025

**Fig. 7:**
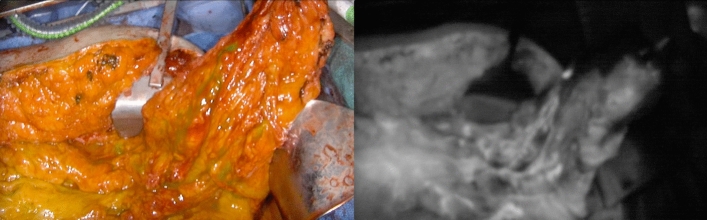
Perfusion assessment of an omental pedicled flap using indocyanine green (ICG) fluorescence angiography using the Stryker SPT^®^ platform. Images depict white light overlay mode (left panel) and fluorescence grayscale mode (right panel). Note adequate perfusion of the tip of the flap, which was supplied only by the right gastroepiploic artery. © Cleveland Clinic Foundation, 2025

An omental pedicled flap (Fig. 6) is based on one of the gastroepiploic pedicles [29]. Perfusion to the distal flap may be confirmed using indocyanine green (ICG) fluorescence angiography (Fig. 7). Slooter et al. demonstrated a significant (50%) decrease in perineal wound dehiscence in flaps confirmed to be well perfused with ICG (22% vs. 42%, *p* = 0.05) [30, 31].

In severe cases with extensive fibrotic destruction of the perineum (i.e., watering-can perineum), myocutaneous flaps, such as bilateral gluteus maximus V–Y advancement or gracilis muscle flaps, may be considered for achieving perineal soft tissue coverage. These reconstructive options are typically performed in collaboration with plastic surgery colleagues.

### Incisional negative pressure wound therapy

**Fig. 8:**
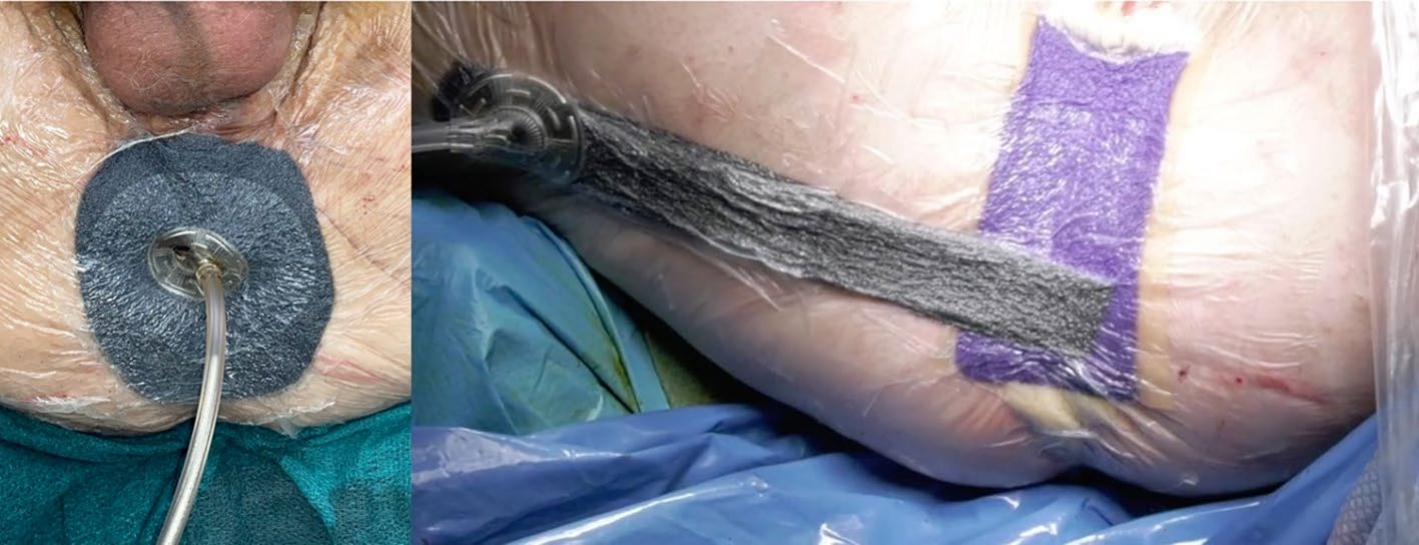
Incision negative pressure wound therapy (iNPWT) including Solventum 3 M V.A.C.^®^ Peel and Place (left panel) and 3 M Prevena™ (right panel)—note the use of karaya stoma adhesive paste posteriorly and anteriorly to provide a seal in the midline and the umbilical cord so that the phalange is offloaded laterally for patient comfort. © Cleveland Clinic Foundation 2025

After closure of the perineal skin, a short-term (1-week) incisional negative pressure wound therapy device (i-NPWT) may be applied [32]. Alternatively, the skin may be left open, and a traditional wound vacuum may be placed. Stoma adhesive (karaya) paste is used to obtain a seal in the intergluteal cleft (Fig. 8).

### Treatment of perineal wound dehiscence

Early, aggressive, multimodal treatment of perineal wound dehiscence and/or surgical site infections helps prevent wound fibrosis and epithelization [33, 34].

### Future directions

Just as the current strategy focuses on prevention and treatment of class 4 perianal disease, future research should follow the same pattern. In particular, the biological preparation of the perineum for successful closure remains poorly understood. Surgical factors (such as performing TME rather than close rectal dissection) and interventions for NHPW are easier to study. However, the relative rarity of this situation, even in high-volume units, requires a collaborative research approach. Class 4 disease is a key area of study for the TOpClass consortium.

## Conclusions

Non-healing perineal wounds after elective ISP for pCD remain a common and morbid complication. A pragmatic approach to preoperative optimization, combined with relatively simple and readily available surgical techniques, may help prevent complications.

## Data Availability

The data used in this study are not publicly available but are available upon reasonable request.
